# The Rapalogue, CCI-779, Improves Salivary Gland Function following Radiation

**DOI:** 10.1371/journal.pone.0113183

**Published:** 2014-12-01

**Authors:** Maria Morgan-Bathke, Zoey I. Harris, Deborah G. Arnett, Rob R. Klein, Randy Burd, David K. Ann, Kirsten H. Limesand

**Affiliations:** 1 Department of Nutritional Sciences, University of Arizona, Tucson, Arizona, United States of America; 2 Department of Pathology, University of Arizona, Tucson, Arizona, United States of America; 3 Department of Molecular Pharmacology, Beckman Research Institute, City of Hope, Duarte, California, United States of America; National Institute of Dental and Craniofacial Research, United States of America

## Abstract

The standard of care for head and neck cancer typically includes surgical resection of the tumor followed by targeted head and neck radiation. However depending on tumor location and stage, some cases may not require surgical resection while others may be treated with chemoradiation. Unfortunately, these radiation treatments cause chronic negative side effects for patients. These side effects are associated with damage to surrounding normal salivary gland tissue and include xerostomia, changes in taste and malnutrition. The underlying mechanisms of chronic radiation-induced salivary gland dysfunction are unknown, however, in rodent models persistently elevated proliferation is correlated with reduced stimulated salivary flow. The rapalogue, CCI-779, has been used in other cell systems to induce autophagy and reduce proliferation, therefore the aim of this study was to determine if CCI-779 could be utilized to ameliorate chronic radiation-induced salivary gland dysfunction. Four to six week old *Atg5^f/f^; Aqp5-Cre*, *Atg5^+/+^; Aqp5-Cre* and FVB mice were treated with targeted head and neck radiation. FVB mice were treated with CCI-779, chloroquine, or DMSO post-radiation. Stimulated salivary flow rates were determined and parotid and submandibular salivary gland tissues were collected for analyses. Mice with a defect in autophagy, via a conditional knockout of *Atg5* in the salivary glands, display increased compensatory proliferation in the acinar cell compartment and hypertrophy at 24-72 hours following radiation. FVB mice treated with post-therapy CCI-779 have significant improvements in salivary gland physiology as determined by stimulated salivary flow rates, proliferation indices and amylase production and secretion. Consequently, post-radiation use of CCI-779 allows for improvement of salivary gland function and reestablishment of glandular homeostasis. As CCI-779 is already FDA approved for other uses, it could have a secondary use to alleviate the chronic side effects in head and neck cancer patients who have completed anti-tumor therapy.

## Introduction

Head and neck cancer is one of the most common cancers worldwide. In 2012, about 52,000 new cases were diagnosed in the United States and of these, about 11,500 patients will die from the disease [1]. The current standard of care for head and neck cancer includes surgical resection of the tumor followed by radiation and chemotherapy [2]. However, less advanced cases may not require surgical resection. The course of treatment for head and neck cancer is determined by evaluating tumor stage and location. Generally, chemoradiation therapy for head and neck cancer consists of radiotherapy combined with cisplatin or radiotherapy combined with cetuximab [3]. Combined chemoradiation therapy is the preferred treatment for locally advanced or inoperable tumors of the head and neck. In addition, this combined therapy increases the 5-year survival rate by 6.5% when compared to radiotherapy alone [4], [5]. Unfortunately, this radiotherapy causes significant negative side effects, both acute and chronic. The acute effects of radiotherapy occur within a few days to weeks following initial treatment and are most likely caused by high levels of acinar cell death [6], [7], [8], [9], [10]. These acute side effects include reduction in saliva production, loss of acinar cells, glandular shrinkage, changes in saliva composition, xerostomia, and mucositis [6], [7], [8], [9], [10], [11]. The chronic effects of radiation are seen months to years following initial treatment and may be caused by persistent compensatory proliferation, vascular damage and parenchymal cell loss [7], [8], [11], [12], [13]. The chronic side effects include decreased salivary output, reduction in acinar cells, accumulation of fibrotic tissue, and increased rates of dental caries [8], [14], [15]. These negative side effects can last for the remainder of a patient's life and greatly diminish their quality of life while increasing their financial burden [16].

Autophagy is a homeostatic process that is constitutively active in essentially all tissues to play a “housekeeping role” by removing damaged or misfolded proteins [17], [18]. Based on this housekeeping role, autophagy could be utilized following targeted head and neck radiation to reverse chronic salivary gland dysfunction by allowing for removal of damaged proteins and organelles. Induction of autophagy and inhibition of proliferation are proposed methods of action of rapamycin or rapalogues. Rapamycin induces macroautophagy (hereafter referred to as autophagy) through the inhibition of mTOR complex 1 (mTORC1) which is the complex of mTOR with Raptor [19]. Rapamycin is an immunosuppressant that was originally used in transplant patients and it is currently approved by the FDA for its use in renal cell carcinoma and mantle cell lymphoma [20]. In addition, rapalogues are currently being tested in clinical trials for a variety of other cancer types [21]. Rapamycin inhibits mTORC1 by interacting with immunophilin FKBP-12 to form an inhibitory complex. The rapamycin-FKBP-12 complex binds and sequesters Raptor so that it is unable to activate mTOR. Inhibition of mTORC1 allows for the induction of autophagy as mTORC1 inhibits the initiation of autophagy through sequestration of autophagy related gene 13 (Atg13) [22]. In addition, this inhibition of mTOR via rapamycin allows for the inhibition of aberrant proliferation by downregulation of p70S6K. Increased proliferation in the acinar cells of parotid salivary glands at chronic time points following targeted head and neck radiation is associated with decreased total salivary gland function [8], [23]. Therefore, rapamycin could be utilized to inhibit this proliferative response.

Autophagy is considered a “double-edged sword” of cancer and cancer therapeutics (reviewed in [24] and [25]). Therefore, we also utilized an autophagy inhibitor, chloroquine (CQ) in the current study. CQ is an antimalarial drug that enters the lysosome. Once inside the lysosome it becomes protonated and therefore raises the lysosomal pH which leads to an inhibition of lysosomal activity [26].

We utilized the *Atg5^f/f^:Aqp5-Cre* mouse model which have a conditional knockout of *Atg5*, a gene necessary for autophagy to occur, in the salivary glands (parotid and submandibular) [27]. Therefore, these mice have “autophagy-deficient” salivary glands and we have previously shown that these mice have an exacerbated acute response to radiation resulting in elevated apoptosis and further declines in stimulated salivary flow rates when compared to wildtype controls [28]. Previous mouse model studies have shown that chronic loss of salivary gland function after radiation correlates with elevated proliferation in the acinar compartment (as determined by PCNA staining) and decreased expression of a key salivary protein, amylase, in the parotid gland [29]. We aimed to determine if a lack of autophagy affected the chronic response of the submandibular and parotid glands to targeted head and neck radiation and if the use of rapalogues could be utilized to improve total salivary gland function following radiotherapy. We hypothesize that CCI-779 could have a secondary clinical use in improving total salivary gland function following targeted head and neck radiation.

## Materials and Methods

### Ethics Statement

All animals were housed and treated in accordance with the University of Arizona Institutional Animal Care and Use Committee (IACUC). All animals were sacrificed using Avertin injections followed by exsanguination through an incision of the renal artery and vein. All experiments were approved by IACUC.

### Production of Atg5^f/f^;Aqp5-Cre Mice

The lineage and baseline characteristics of these mice have been described previously [27]. Briefly, a mouse line that contained a floxed *Atg5* gene (*Atg5^f/f^*) was crossed with an *Aqp5-Cre* mouse line to produce *Atg5^f/f^;Aqp5-Cre* and *Atg5^+/+^;Aqp5-Cre* mice. The genotype of each mouse was determined using DNA isolated from tail tips. 1 µl of isolated DNA and 2 µl of Atg5 or Cre recombinase primer (Integrated DNA Technologies (IDT) Coral Ville, IA) were added to Hot Start PCR Mix Tubes (Bioneer Alameda, CA). The Hot Start PCR Mix Tubes were then placed into a thermal cycler for 2 hours (Bio-Rad Hercules, CA). The PCR product was loaded into an agarose gel with ethidium bromide and run at 150 volts for about 30 minutes. The VersaDoc Imaging System (Bio-Rad) was used to image the ethidium bromide gels.

### Radiation Treatment

Four to six week old FVB, *Atg5^f/f^;Aqp5-Cre*, or *Atg5^+/+^;Aqp-Cre* mice were sedated using intramuscular injections of a ketamine/xylazine mixture (50 mg/kg and 10 mg/ml respectively) (Western Medical Supply Arcadia, CA). Following sedation, mice were constrained in a 50 ml conical tube and the head and neck region was exposed to a single 5-Gy dose of radiation (^6^°Co therapeutic irradiator, Theratron-80, Atomic Energy of Canada Ltd., Ottawa, Canada). The rest of the body was shielded with>6 mm lead to prevent systemic side effects. Mice were monitored during radiotherapy through the use of an in chamber video camera.

### Histology

Salivary gland tissue (parotid, submandibular, and sublingual) was excised and fixed in 10% formalin (Fisher Scientific Waltham, MA) for 24 hours and then placed into 70% ethanol. Tissues were paraffin embedded, serial sectioned (4 µm) and one slide was stained with hematoxylin and eosin (H&E) by the Histology Service Laboratory in the Department of Cellular and Molecular Medicine at the University of Arizona. H&E stained slides were analyzed by a clinical pathologist (RRK) and the senior author (KHL). Total acinar cell counts were obtained by quantifying the number of acini present in the parotid glands from three fields of view/animal. N = 4/treatment. Data are reported as the mean ±SEM.

### PCNA Staining

The serial sectioned unstained slides from the parotid gland were baked for 45 minutes at 37°C. The slides are then rehydrated in Histoclear, 100% ethanol, 95% ethanol, 70% ethanol, 50% ethanol and water for 10 minutes each. Antigen retrieval was achieved with citric acid (0.01 M). Next, slides were blocked in ABC Rabbit Kit (Vector Laboratories Burlingame, CA) and incubated in Proliferating Cellular Nuclear Antigen (PCNA) antibody (Santa Cruz Biotechnology Dallas, TX) at a 1∶1000 dilution in PBS overnight at 4°C. The next day slides were washed in PBS and then in 1% hydrogen peroxide for 5 minutes each. Slides were then incubated in biotinylated secondary antibody (ABC Rabbit Kit, Vector Laboratories) for 50 minutes at room temperature. Then, slides were washed in PBS and incubated in ABC reaction kit (Vector Laboratories) for 30 minutes. Next, slides were washed in PBS and incubated in 3, 3′ Diaminobenzidine (DAB) for 6 minutes. Water was used to stop the DAB reaction and slides were stained in hematoxylin for about 2 seconds and slides were dehydrated using 50% ethanol, 70% ethanol, 95% ethanol, 100% ethanol, and Histoclear for 10 minutes each. Coverslips were mounted over tissue using Protocol Securemount (Fisher Scientific). The stained tissue sections were visualized using light microscopy and images (20X) were taken with a Leica DM5500 (Leica Microsystems Wetzlar, Germany) and a 4-megapixel Pursuit camera (Diagnostic Instruments, Inc Sterling Heights, MI). PCNA-positive cells in major ductal structures were quantified separately from the remaining gland (termed the acinar compartment). The total number of cells counted from the ductal compartment was ∼75/image and the acinar compartment was ∼750/image. Percent positive cells were determined by averaging the number of positive cells/total number of cells from a specific glandular compartment using a minimum of three fields of view per animal. N = 4/treatment group.

### Western Blotting

Parotid glands were excised and homogenized in RIPA buffer with 5 mM sodium orthovanadate (Fisher Scientific), protease inhibitor cocktail (Sigma-Aldrich) and 100 mM PMSF (Fisher Scientific). The samples were then boiled for 10 minutes and sonicated until homogenous. 100 mg of each protein sample was added to 12% polyacrylamide gels and transferred to 0.45 µm Immobilon-P membranes (Millipore Billerica, MA). Membranes were blocked using either non-fat dry milk or 5% BSA then immunoblotted with one of the following antibodies: anti-ERK (Promega Madison, WI), anti-pAkt (Cell Signaling) and anti-pS6K (Cell Signaling). ECL substrate (Fisher Scientific) was used as instructed by the manufacturer for detection. The membranes were stripped using Restore Western Blotting Stripping buffer (Fisher Scientific) and then blocked and re-probed as described above.

### Densitometry

Black and white images of Western membranes were imported into Image J software for quantification of bands as per the Image J Software Guide [30]. Quantified bands from 3-4 animals per treatment group and time point were then normalized to their loading control and displayed as a ratio to that loading control.

### CCI-779, DMSO, and Chloroquine

Targeted head and neck radiation was given on day 0. Mice were injected intraperitoneally (*i.p.*) with 10 nM CCI-779 (Wyeth Pharmaceuticals New York, NY) diluted in DMSO, or DMSO as a vehicle control on days 4-8 following radiotherapy, resulting in a total dose of 40 mg/kg dose throughout the course of treatment. This dose of CCI-779 is similar to doses used in other pre-clinical models and did not result in toxicity. For hydroxychloroquine sulfate (CQ) (Sigma-Aldrich) treatment, mice were given i.p injections of CQ (30 mg/kg) diluted in sterile PBS on days 4-8 following radiation.

### Saliva Collection

Mice received an injection (*i.p.*) of carbachol (0.25 mg/kg body weight) and saliva was collected by vacuum aspiration from each mouse for 5 minutes immediately following this injection. Saliva was collected into pre-weighed tubes and immediately placed on ice. Salivary flow rates were normalized to the average of the unirradiated (UT) group for each respective treatment group or genotype. N≥10/treatment group.

### Saliva Composition

Total protein composition of saliva was determined using the Bio-Rad Experion System. Samples were treated and analyzed for amylase concentration according to the Experion Pro260 Analysis Kit protocol. N≥10/treatment group.

### Amylase Area Staining

Serial sectioned unstained slides from parotid glands were dehydrated and antigen retrieval was performed as described above for PCNA. 0.5% NEN was used to block slides at room temperature for 1 hour and they were incubated in anti-amylase primary antibody at a 1∶500 dilution (Sigma Aldrich St. Loius, MO) overnight at 4°C. Slides were washed with PBS and incubated in anti-rabbit Cy2-conjugated secondary antibody (1∶500) (Invitrogen Grand Island, NY) at room temperature for 1 hour. Next, the slides were counterstained with DAPI and mounted with 50% glycerol in 10 mM Tris-HCl. Fluorescent images were visualized on a Leica DM5500 Microscope System and digitally captured with a Pursuit 4 Megapixel CCD camera using Image Pro 7.0 software and morphometric analysis was performed with ImagePro 7.0 (Media Cybernetics Rockville, MD). Twenty fields of view (FOV = 0.39 mm^2^) per mouse were used to determine positive amylase area. Amylase area is expressed as the percentage of tissue area stained positive for amylase to the total area of the parotid gland and the threshold fluorescence range was equivalent for all slides imaged. N = 4/treatment group.

### Statistical Analysis

Data are reported as the mean ± the SEM. All statistical analyses were conducted using a one-way analysis of variance (ANOVA) followed by a Bonferroni post-hoc test performed using InStat GraphPad 3 software (San Diego, CA). Salivary flow rates were standardized to unirradiated (UT) groups before the statistical analysis was performed. For densitometry analysis of immunoblotting, each treatment group was compared to one another by each treatment day separately. Treatment groups with the same letter are not statistically different from one another.

## Results

### Atg5^f/f^;Aqp5-Cre autophagy-deficient mice display increased hyperplasia and elevated compensatory proliferation following targeted head and neck irradiation

**Figure 1:**
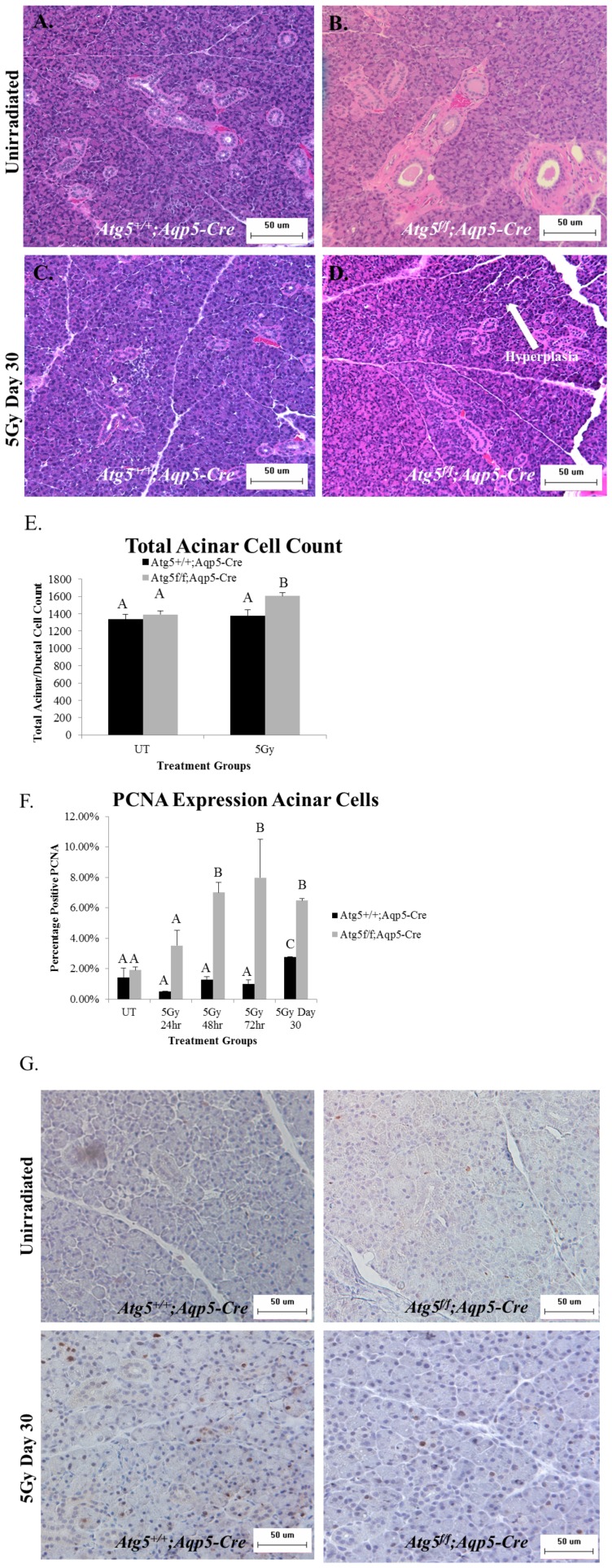
*Atg5^f/f^;Aqp5-Cre* autophagy-deficient mice display increased hyperplasia and elevated compensatory proliferation of the parotid acinar cell population following targeted head and neck irradiation. *Atg5^+/+^;Aqp5-Cre* and *Atg5^f/f^;Aqp5-Cre* (autophagy-deficient) mice were treated with a single 5-Gy dose of targeted head and neck radiation on day 0. At 24, 48, 72 hours and day 30 following treatment, salivary tissue was collected and serial sections were stained. Letters above treatment groups are used to signify statistical significance; treatment groups with the same letters are not significantly different from each other. Significant differences (*p*<0.05) were determined using an ANOVA followed by a post-hoc Bonferroni multiple-comparison test. Data are presented as the mean ±SEM. **A.**) Representative image of H&E staining of unirradiated *Atg5^+/+^;Aqp5-Cre* control-mice. **B.**) Representative image of H&E staining of *Atg5^f/f^*;*Aqp5-Cre* unirradiated mice. **C.**) Representative image of H&E staining of irradiated *Atg5^+/+^;Aqp5-Cre* mice 30 days after radiotherapy. **D.**) Representative image of H&E staining of irradiated *Atg5^f/f^*;*Aqp5-Cre* mice 30 days after radiotherapy. **E.**) Total acinar cell counts taken from 3–5 images per mouse 30 days after radiotherapy. *p*<0.05; n≥4 per genotype. UT: unirradiated. **F.**) Serial sections were stained for PCNA, a marker of proliferation, and the graph represents the number of acinar cells with positive PCNA staining in the parotid glands as a percentage of the total number of acinar cells. *p*<0.05, n = 4 per genotype/per treatment. **G.**) Representative images of positive PCNA staining in the parotid glands.

*Atg5^f/f^;Aqp5-Cre* autophagy-deficient mice, which have a conditional knockout of *Atg5*in the Aqp5-expressing cells of the salivary glands (parotid and submandibular), were utilized to determine the effect of the lack of autophagy on parotid and submandibular salivary gland structure following targeted head and neck radiation. We have previously shown that these *Atg5^f/f^;Aqp5-Cre* mice lack the Atg5 protein in the parotid and submandibular salivary glands [31]. H&E stained slides from irradiated *Atg5^+/+^;Aqp5-Cre* mice and *Atg5^f/f^;Aqp5-Cre* autophagy-deficient mice were compared and evaluated by a clinical pathologist. We aimed to determine if a conditional inactivation of autophagy resulted in varied structural changes of the salivary glands following irradiation. Evaluation of H&E stained salivary gland tissues (parotid and submandibular) at 30 days following irradiation (representative images shown in Figure 1A–D) reveals that parotid salivary glands from *Atg5^f/f^;Aqp5-Cre* autophagy-deficient mice have a slight increase in hyperplasia when compared to *Atg5^+/+^;Aqp5-Cre* mice (shown with arrows in Figure 1D), which was confirmed by enumerating the total number of nuclei in the acinar compartment of the parotid glands (Figure 1E). However, hyperplasia was not present in the submandibular glands (data not shown).

Rodent models have previously demonstrated that increased rates of parotid acinar cell proliferation following targeted head and neck radiation (days 9–90) are correlated with poor total salivary gland function [29]. We therefore, utilized proliferating cellular nuclear antigen (PCNA) to assess rates of compensatory acinar cell proliferation in the parotid glands following radiation. At 24 hours following radiation, both *Atg5^f/f^;Aqp5-Cre* autophagy-deficient and *Atg5^+/+^;Aqp5-Cre* mice displayed PCNA levels similar to those in unirradiated (UT) mice (Figure 1F). However, starting at 48 hours following irradiation, *Atg5^f/f^;Aqp5-Cre* autophagy-deficient mice exhibited a significant (*p*<0.05) 7% increase in PCNA level when compared to both unirradiated (UT) and irradiated *Atg5^+/+^;Aqp5-Cre* mice, and this continued at 72 hours post-irradiation (Figure 1F). At 30 days following radiation, the parotid glands of *Atg5^f/f^;Aqp5-Cre* autophagy-deficient mice continued to exhibit significantly increased (*p*<0.05) levels of PCNA (6.5%) (Figure 1F). The *Atg5^+/+^;Aqp5-Cre* mice also have significantly increased levels of PCNA (2.7%) at the 30 day time point when compared to unirradiated mice; however this increase was significantly lower than the autophagy-deficient mice. These analyses illustrate that the inactivation of autophagy in salivary acinar cells promoted parotid salivary gland hyperplasia in addition to dysregulated cellular proliferation following radiation when compared to irradiated *Atg5^+/+^;Aqp5-Cre* mice. Cumulatively, it appears that lack of autophagy exacerbates the chronic response of the parotid salivary glands to targeted head and neck radiation, as displayed by increased levels of acinar cell proliferation in the autophagy-deficient mice.

### Radiation induces hyperactivation of the mTOR pathway 4–5 days following treatment

**Figure 2:**
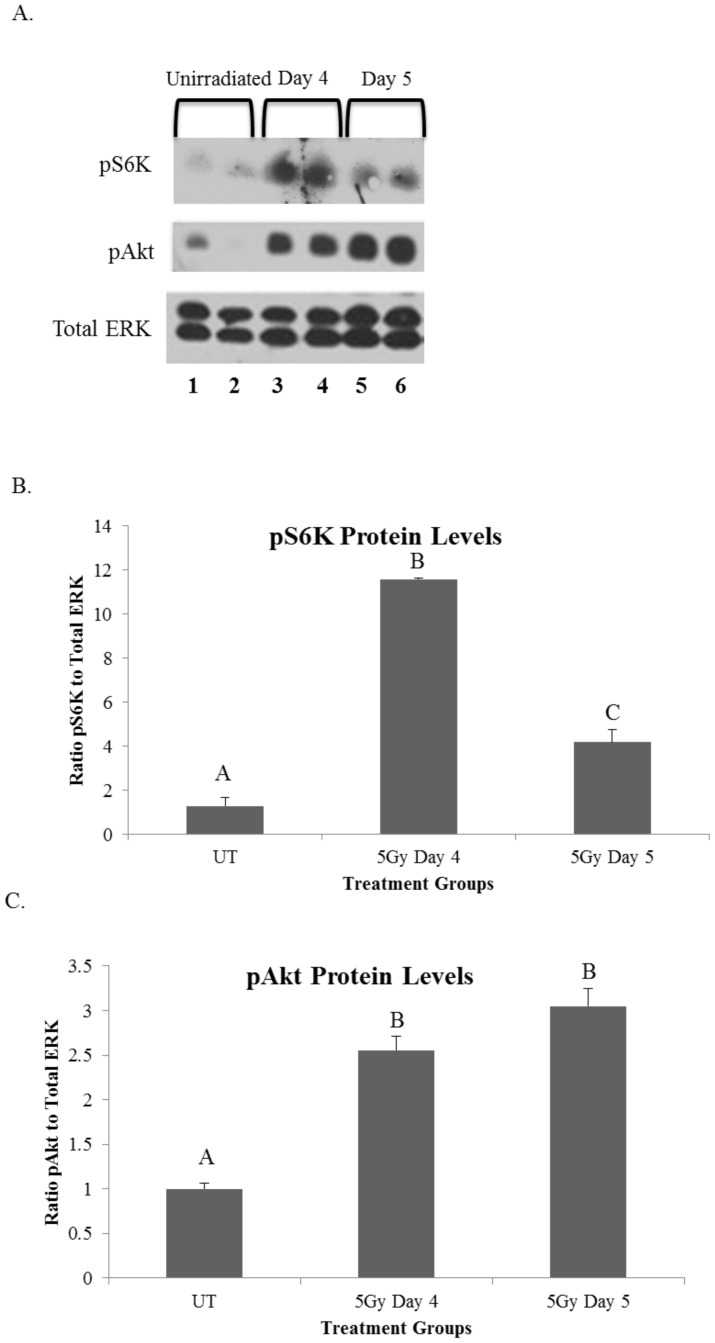
Radiation induces hyperactivation of the mTOR pathway 4–5 days following treatment. On day 0 FVB wild-type mice were either unirradiated or treated with a single 5-Gy dose of targeted head and neck radiation. Letters above treatment groups are used to signify statistical significance; treatment groups with the same letters are not significantly different from each other. Significant differences (*p*<0.05) were determined using an ANOVA followed by a post-hoc Bonferroni multiple-comparison test. Data are presented as the mean ±SEM. **A**.) Parotid glands were collected at respective time points and protein was isolated to be used in immunoblots (representative blot shown) and probed for pS706K (top panel), pAkt (middle panel) or total ERK (bottom panel). **B**.) The graph represents the ratio of phosphorylated S6K to total ERK, which was quantified using densitometry. *p*<0.05, n≥3 per time point/treatment. UT: unirradiated. **C**.) The graph represents the ratio of phosphorylated Akt to total ERK, which was quantified using densitometry. *p*<0.05, n≥3 per time point/treatment. UT: unirradiated.

Next, we aimed to determine if activation of the mTOR pathway was elevated following targeted head and neck radiation as this pathway has an intricate relationship with proliferation indices and autophagy. In addition, Bozorgi et al recently showed that activation of the mTOR pathway occurs with salivary gland damage associated with ductal ligation [31] and mTOR can be inactivated pharmacologically with rapalogue (CCI-779) treatment. In the current study, protein was isolated from parotid salivary glands of mice treated with a single 5-Gy dose of targeted head and neck radiation at days 4 and 5 following treatment. These time points were chosen due to the significant decrease in total salivary gland function at day 3 post radiation that remains depressed at chronic time points [8]. Protein levels of both phosphorylated S6K (p70S6K) and phosphorylated Akt (pAkt) were measured as these proteins play a key role in the mTOR pathway [32]. Akt is phosphorylated upstream of mTORC1 activation and p70S6K is a downstream target of activated mTORC1. Therefore, an increase in these proteins suggests an increase in mTOR activity. P70S6K is increased on days 4 and 5 following targeted head and neck radiation when compared to unirradiated (UT) controls (Figures 2A and B). Phosphorylation of Akt on serine^473^ (pAkt) was also significantly increased from unirradiated (UT) in irradiated samples at both days 4 and 5 following radiotherapy (Figures 2A and C). These results suggest hyperactivation of the mTOR pathway following targeted head and neck radiation occurs at similar time points as the acute loss of salivary gland function and could play a role in establishing chronic loss of function.

### Post-therapy treatment with the rapalogue, CCI-779, allows for improvement of salivary gland function at chronic time points following targeted head and neck radiation in FVB mice, while radiation with post-therapy chloroquine (CQ) treatment in FVB mice does not improve salivary flow rates

**Figure 3:**
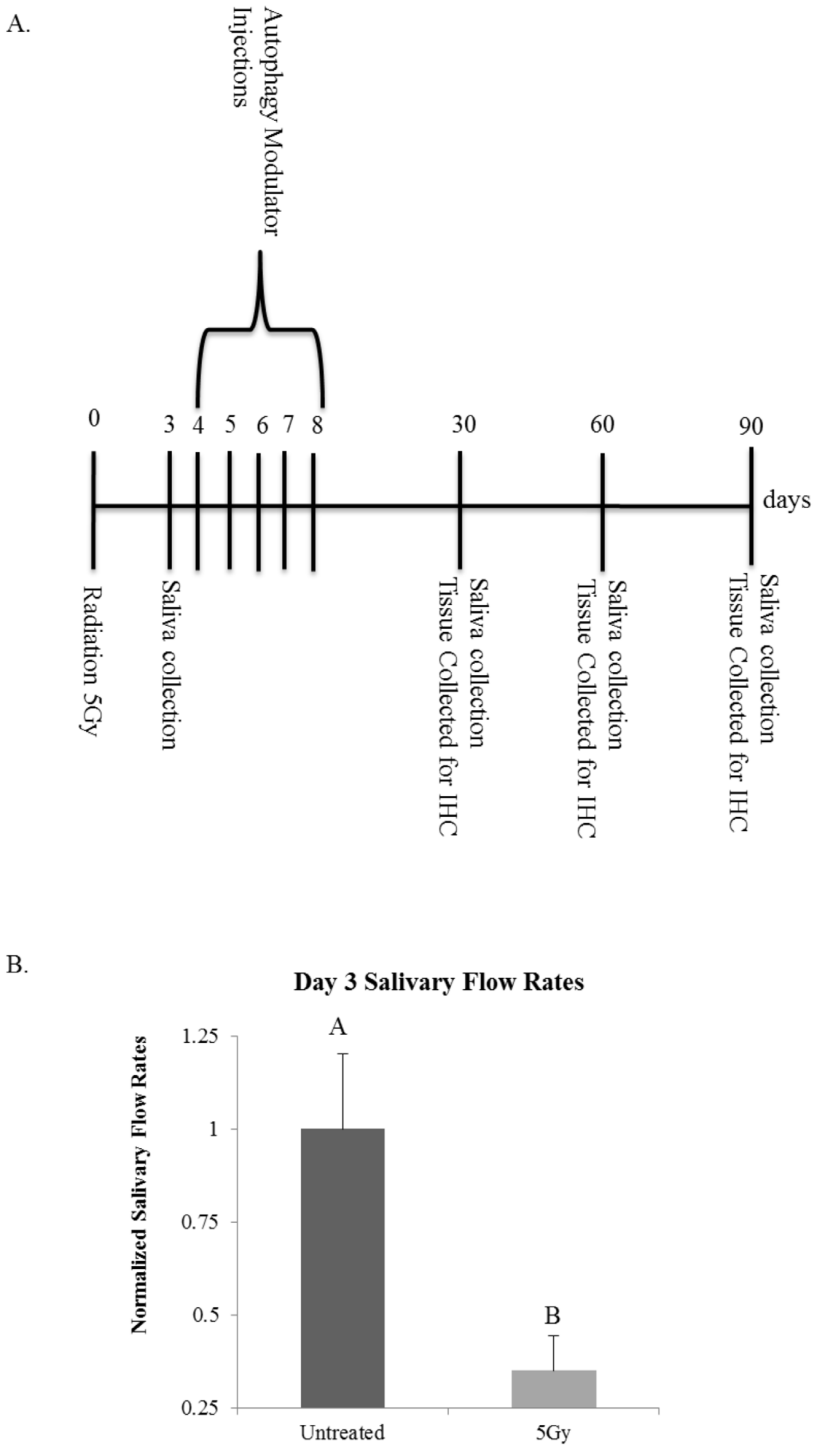
Experimental Design. **A**.) Experimental Setup for CCI-779 or CQ injections and tissue collection for analysis. **B**.) On day 0 FVB wild-type mice were either unirradiated or treated with a single 5-Gy dose of targeted head and neck radiation. Letters above treatment groups are used to signify statistical significance; treatment groups with the same letters are not significantly different from each other. Significant differences (*p*<0.05) were determined using an ANOVA followed by a post-hoc Bonferroni multiple-comparison test. Data are presented as the mean ±SEM. Stimulated salivary flow rates were determined as described in Materials and Methods section on day 3. N≥10/treatment group.

**Figure 4:**
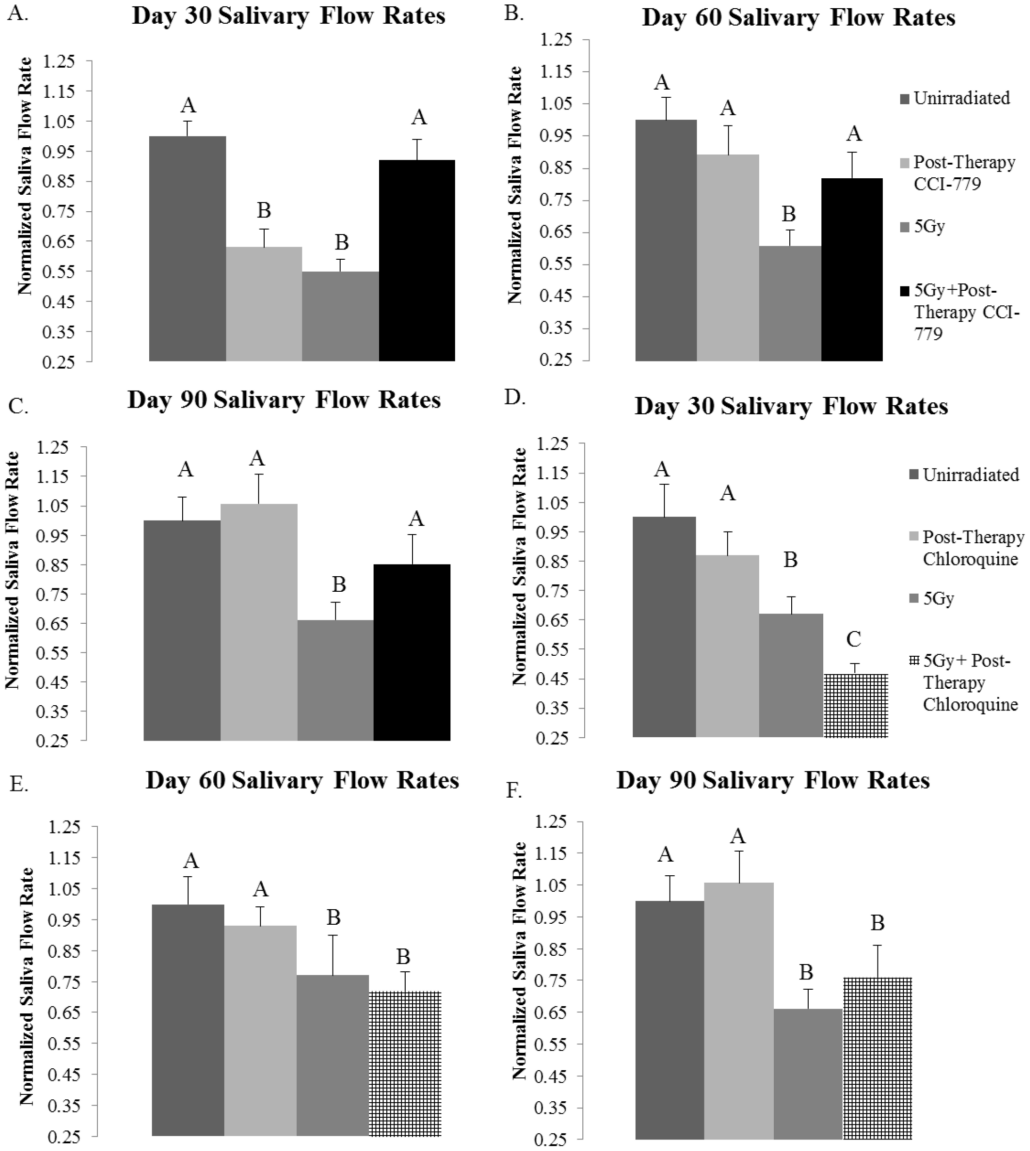
Post-therapy CCI-779 allows for improvement of salivary flow rates, while post-therapy chloroquine (CQ) does not have a similar effect. The head and neck region of FVB mice was exposed to a single 5-Gy dose of radiation and mice received injections of vehicle, CCI-779 or CQ on days 4–8 following the initial radiation treatment. Irradiated flow rates were normalized to corresponding unirradiated controls after the radiation treatment, n≥10 per treatment group. Significant differences (*p*<0.05) were determined using an ANOVA followed by a post-hoc Bonferroni multiple-comparison test. Letters above treatment groups are used to signify statistical significance; treatment groups with the same letters are not significantly different from each other. Data are presented as the mean ±SEM. **A.**) Day 30 post-therapy CCI-779. **B.**) Day 60 post-therapy CCI-779. **C.**) Day 90 post-therapy CCI-779. **D.**) Day 30 post-therapy CQ. **E.**) Day 60 post-therapy CQ. **F.**) Day 90 post-therapy CQ.

We examined the effect of CCI-779, a rapalogue that induces autophagy, down regulates proliferation and inhibits the mTOR signaling pathway, on total salivary gland function following radiation. In comparison, we also examined the effectiveness of chloroquine (CQ), an autophagy inhibitor that has the opposite mechanism of action as rapalogues. Mice were treated with targeted head and neck radiation on day 0. On day three, saliva was collected to ensure that significant decreases in total salivary gland output had occurred (Figure 3B). On days 4–8 mice received injections (*i.p*.) of CCI-779, CQ, or DMSO as a vehicle control (Figure 3A). Saliva was again collected on days 30, 60, and 90 following radiation treatment. Irradiated mice had significantly decreased salivary flow rates when compared to unirradiated (UT) mice at all of the time points evaluated (Figures 4A–F). However, the salivary output of mice that were treated with radiation plus post-therapy CCI-779 did not differ from mice that were unirradiated (UT) at any of the time points evaluated (Figures 4A–C). At day 30, mice treated with radiation and post-therapy CQ exhibited significantly decreased salivary flow rates from both unirradiated (UT) and irradiated mice (Figure 4D). At days 60 and 90 following targeted head and neck radiation, irradiated mice with post-therapy CQ continued to demonstrate significantly decreased stimulated salivary flow rates when compared to unirradiated (UT) (Figures 4E and F). These results show that post-therapy treatment with CCI-779 allows for significantly improved stimulated salivary flow rates at days 30, 60, and 90 following radiotherapy. Post-therapy CQ treatment was unable to improve salivary gland function throughout the time points evaluated.

### Histological changes in parotid and submandibular salivary glands following radiation and CCI-779 administration

**Figure 5:**
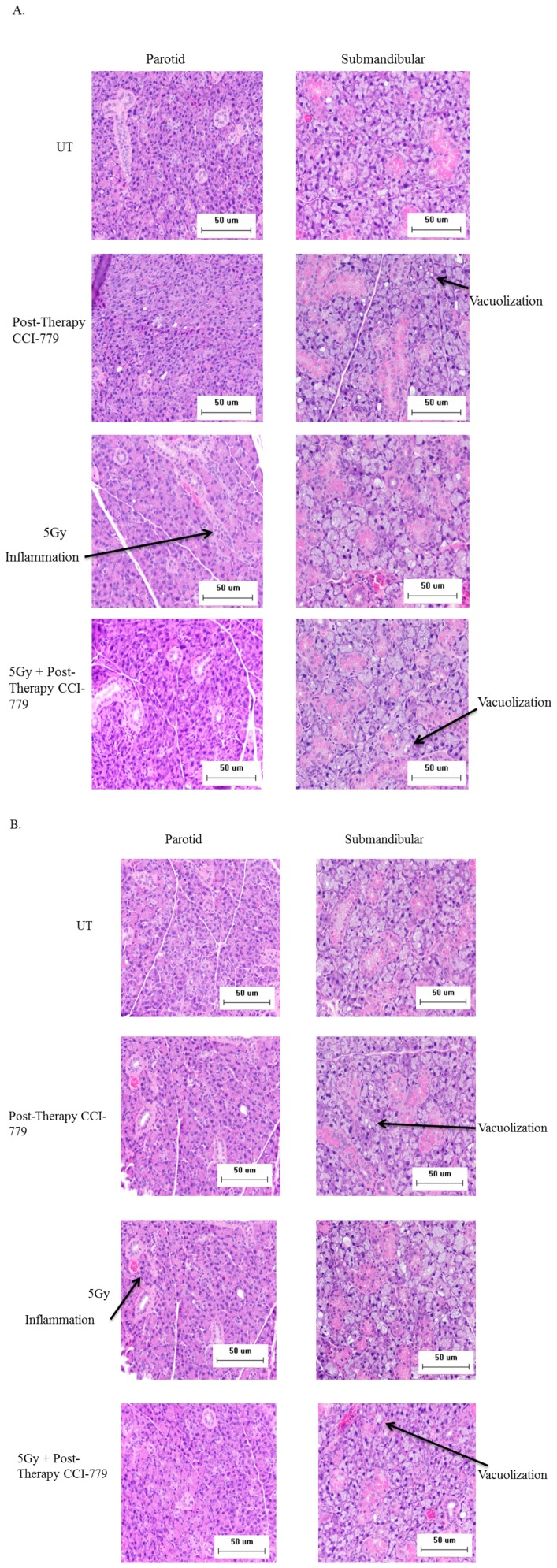
Histological changes in parotid and submandibular salivary glands structure following radiation and CCI-779 administration. The head and neck region of FVB mice was exposed to a single 5-Gy dose of radiation and mice received injections of DMSO vehicle or CCI-779 on days 4-8 following initial radiation. UT: unirradiated. **A.**) Representative H&E sections were analyzed for differences in salivary gland structure 30 days following radiotherapy. **B.**) Representative H&E sections were analyzed for differences in salivary gland structure 90 days following radiotherapy.

Analysis of day 30 and day 90 H&E stained slides revealed that mice treated with CCI-779 alone, radiation with post-therapy DMSO, and radiation with post-therapyCCI-779 had increased vacuolization within the submandibular glands when compared to unirradiated (UT) mice (marked with arrows in Figure 5A–B). Increased vacuolization was not observed in the parotid glands at either time point. At day 30 irradiated mice had signs of mild inflammation in the periductal region of the parotid glands, but not the submandibular, and this inflammation persisted out to day 90 (Figures 5A and B). These pathology changes were not observed in the parotid or submandibular salivary glands of the unirradiated, CCI-779 alone, or radiation with post-therapy CCI-779 groups. These data suggest that the characteristic histological changes in the parotid salivary glands caused by radiation treatment appear to be improved in mice treated with post-therapy CCI-779.

### Treatment of FVB mice with radiation plus post-therapy CCI-779 improves parotid acinar cell proliferation indices and amylase production to levels similar to unirradiated (UT) mice

**Figure 6:**
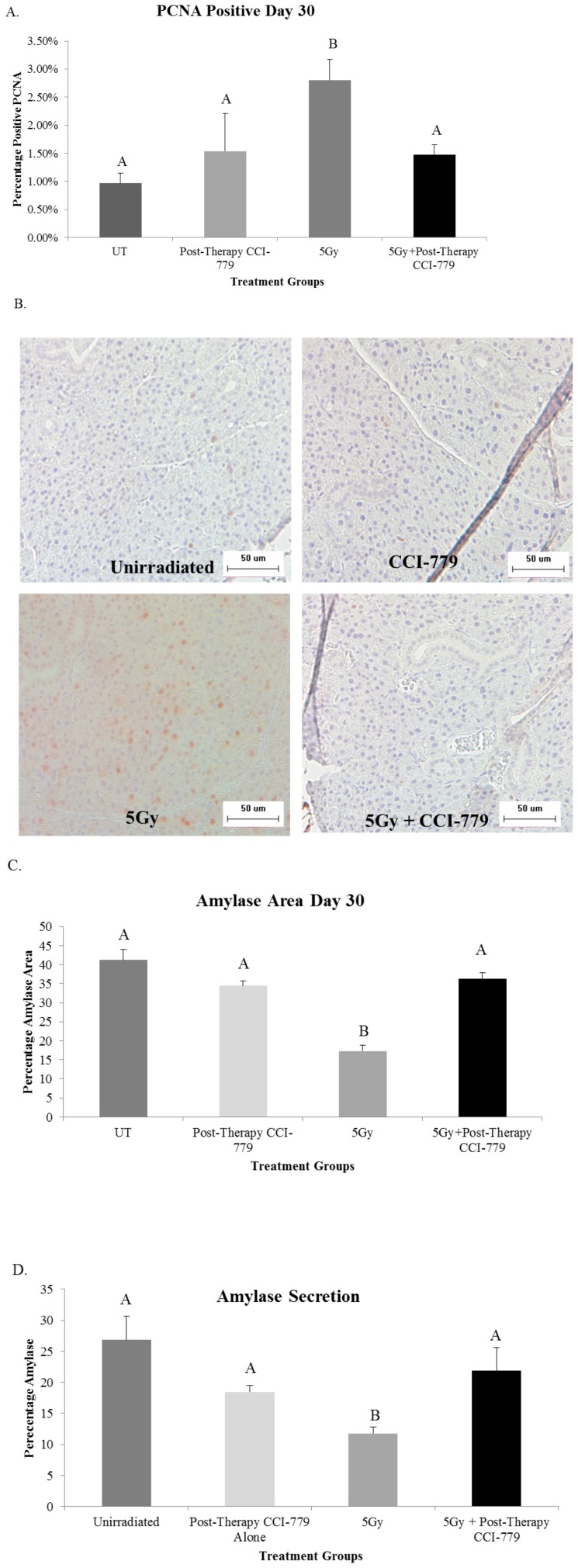
Radiation plus post-therapy CCI-779 improves parotid acinar cell proliferation indices and amylase production levels similar to unirradiated mice. The head and neck region of FVB mice was exposed to a single 5-Gy dose of radiation and mice received injections of vehicle or CCI-779 on days 4-8 following initial radiation treatment. Parotid salivary glands were then collected 30 days following treatment. Significant differences (*p*<0.05) were determined using an ANOVA followed by a post-hoc Bonferroni multiple-comparison test. Letters above treatment groups are used to signify statistical significance; treatment groups with the same letters are not significantly different from each other. Data are presented as the mean ±SEM. UT: unirradiated. **A.**) Serial sections were stained for PCNA, a marker of proliferation, and the graph represents the number of acinar cells with positive PCNA staining in the parotid glands as a percentage of the total number of acinar cells. n = 4 per genotype/per treatment. **B.**) Representative images of positive PCNA staining. **C.**) Serial sections were stained to determine positive amylase area of the parotid glands. The graph represents the positive amylase area as a percentage of the parotid area as a whole. n = 4 per genotype/per treatment. **D.**) Stimulated saliva was collected from mice 30 days after treatment with a single 5Gy dose of targeted head and neck radiation and analyzed for total protein content as described in Materials and Methods. The graph represents the percentage of amylase protein (ranging from 50–57 kD); n = 10 per genotype/per treatment.

Treatment with radiation plus post-therapy CCI-779 allowed for improvement in salivary flow rates and we sought to ensure that other markers of salivary gland homeostasis were also improved in this treatment group. The salivary glands are a highly differentiated, slowly proliferating tissue. As mentioned above, increased levels of proliferation at day 30 following targeted head and neck radiation are correlated with poor salivary output, decreased differentiation (as detected by tissue amylase area) and decreased amylase secretion [29]. Most likely an imbalance between proliferation and differentiation exists and this causes the correlation with decreased function. While at day 30, irradiated mice exhibited a significant increase (*p*<0.05) in PCNA levels (2.8%), a marker of proliferation, mice treated with radiation plus post-therapy CCI-779 had PCNA levels (1.4%) unchanged from unirradiated (UT) mice (0.97%) in the parotid glands (Figure 6A). In addition, the parotid tissues of irradiated mice had significant decreases in the area expressing amylase when compared to unirradiated (UT) mice (Figure 6C). This correlated with a significant decrease in the amount of amylase present in whole saliva collected from these animals (Figure 6D). In contrast, mice that received radiation plus post-therapy CCI-779 had parotid amylase area similar to unirradiated (UT) at day 30, which resulted in improved secretion of amylase into saliva (Figures 6C–D). These results indicate that treatment with post-therapy CCI-779 following targeted head and neck radiation allows for improvement of salivary gland physiology.

## Discussion

Radiotherapy for head and neck cancer causes a multitude of negative side effects that impairs the quality of life for head and neck cancer patients. The purpose of this study was to determine if the rapalogue, CCI-779, could improve total salivary gland function following targeted head and neck radiation. We found that 1) radiation with post-therapy CCI-779 treatment improved salivary flow rates; 2) mice with a conditional knockout of autophagy have an exacerbated compensatory proliferation response at chronic time points following targeted head and neck radiation; and 3) post-therapy CCI-779 improved salivary gland homeostasis shown through normalized proliferation and amylase production levels.

Radiation with post-therapy CCI-779 treatment was able to improve stimulated salivary flow rates out to 90 days following initial radiation treatment (Figure 4A–C). Bartolome et al found that radiation with post-therapy rapamycin *in vivo* was able to repair the epithelial layer of the mouth from mucositis [33]. By utilizing primary human keratinocytes they concluded that this protection was via increased mitochondrial superoxide dismutase (MnSOD) and therefore inhibition of reactive oxygen species (ROS)[34]. However, this inhibition of ROS may also be due to induction of autophagy with rapamycin based on the well-defined ability of autophagy to inhibit oxidative stress[35]. Another recent study found that rapamycin treatment was able to temporarily inhibit salivary gland atrophy associated with ligation[36]. While this rapamycin study only found inhibition of atrophy for 5–7 days following ligation, it differs from the study design discussed here as ligation causes continuous stress to the salivary gland tissue while we utilized a single dose of radiation.

It is also important to note that there was a transient decrease in salivary flow rates at day 30 in the CCI-779 alone group (Figure 4A). However, there is no change in amylase secretion or acinar cell proliferation rates in this group. This suggests a transient decrease in saliva secretion with no impact on other markers of parotid salivary gland physiology. Unfortunately, the effect of rapalogues on saliva secretion in unstressed animals remains unknown.

While mice treated with the rapalogue CCI-779 display improved stimulated salivary flow rates, mice treated with the autophagy inhibitor, CQ, had significantly reduced stimulated salivary flow rates at day 30 that did not improve over the time course (Figures 4D–F). Similarly, in a study conducted by Fang et al, rats with liver ischemia were given CQ injections for up to two days and they found that at the late phase following injections (24–48 hours) CQ worsened the liver injury via both an inhibition of autophagy and an increase in apoptotic cell death [36].

We have shown here that a conditional knockout of autophagy in the salivary glands via *Atg5* loss (*Atg5^f/f^;Aqp5-Cre*) results in increased compensatory proliferation in the parotid acinar compartment following targeted head and neck radiation (Figures 1A–G). These mice suffer from increased proliferation rates as early as 24–72 hours following radiotherapy and persists to day 30 (Figure 1F). Radiation-induced acinar cell compensatory proliferation of parotid salivary glands is frequently reported 7–10 days following treatment [8], [37], [38] and remains elevated at later time points (days 30–90) [29]. The evidence of increased acinar cell proliferation is strengthened by the observation that salivary gland tissue collected from *Atg5^f/f^;Aqp5-Cre* autophagy-deficient mice are hyperplasic 30 days following radiation (Figure 1A–B). These results coincide with multiple studies defining autophagy as a mechanism for maintaining homeostasis in almost all eukaryotic tissues (reviewed in [39]). Therefore, autophagy could play a beneficial role in reestablishing salivary gland homeostasis at chronic time points following targeted head and neck radiation.

Radiation with post-therapy CCI-779 treatment not only allows for improvement of stimulated salivary flow rates but also allows for inhibition of acinar cell compensatory proliferation and improvement of amylase differentiation and secretion levels (Figure 6A–C). Previous studies have found that compensatory proliferation in parotid salivary gland tissue at chronic time points following radiation are associated with poor salivary gland function[29]. Our results show that post-therapy treatment with CCI-779 is able to improve salivary homeostasis following radiation via improvement of amylase secretion and inhibition of compensatory proliferation. These results display a critical role for CCI-779 in the management of the cellular response of the salivary glands to radiation. Similarly, Li et al found that inhibition of mTOR following radiation of adult hematopoietic stem and progenitor cells caused inhibition of premature senescence and therefore maintenance of cellular homeostasis in this stressed condition [40].

A majority of our analysis focused on the parotid gland due to its primary function in stimulated salivary flow rates and increased sensitivity to radiation [10], [41], [42]. Interestingly, we noted an increase of vacuolization in the CCI-779 treated as well as irradiated submandibular glands but not the parotid glands. Few studies have evaluated the presence of vacuoles at chronic radiation time points (60–90 days); one study evaluated submandibular salivary glands (doses of 7.5 or 15 Gy) following a single dose of radiation and two studies evaluated parotid glands following fractionated radiation [43], [44], [45]. All of these studies administered higher levels of radiation to the salivary glands than the current study, which suggests higher doses of radiation may be required to induce vacuolization in the parotid gland. It is also known that the formation of vacuoles is characteristic of the autophagic process [46], which may be contributing to the vacuolization in CCI-779 treated mice. Irradiated parotid salivary gland tissues displayed typical inflammatory cell infiltration that was not observed in the submandibular gland. Importantly, post-therapy administration of CCI-779 ameliorated this pathology consistent with its role as an immunosuppressant [47].

We show here that CCI-779 treatment allows for the improvement of normal total salivary gland function. Normalization of acinar cell proliferation indices and autophagy may be the major mechanisms utilized for this improvement of function. These results are significant as rapalogues such as CCI-779 are currently being studied in clinical trials for the treatment of head and neck cancer with radiation [48], [49]. Therefore, it is feasible that rapalogues could be used in the clinic to ameliorate the multitude of negative side effects suffered by these cancer patients following completion of their cancer therapy.

## Supporting Information

Checklist S1 **NC3Rs ARRIVE Guidelines Checklist.**
(PDF)
